# Supplementary figures and images for: Mexican Ganoderma Lucidum Extracts Decrease Lipogenesis Modulating Transcriptional Metabolic Networks and Gut Microbiota in C57BL/6 Mice Fed with a High-Cholesterol Diet
Source: Nutrients. 2020 Dec 24;13(1):38. doi: 10.3390/nu13010038 (PMC7823885; doi:10.3390/nu13010038)

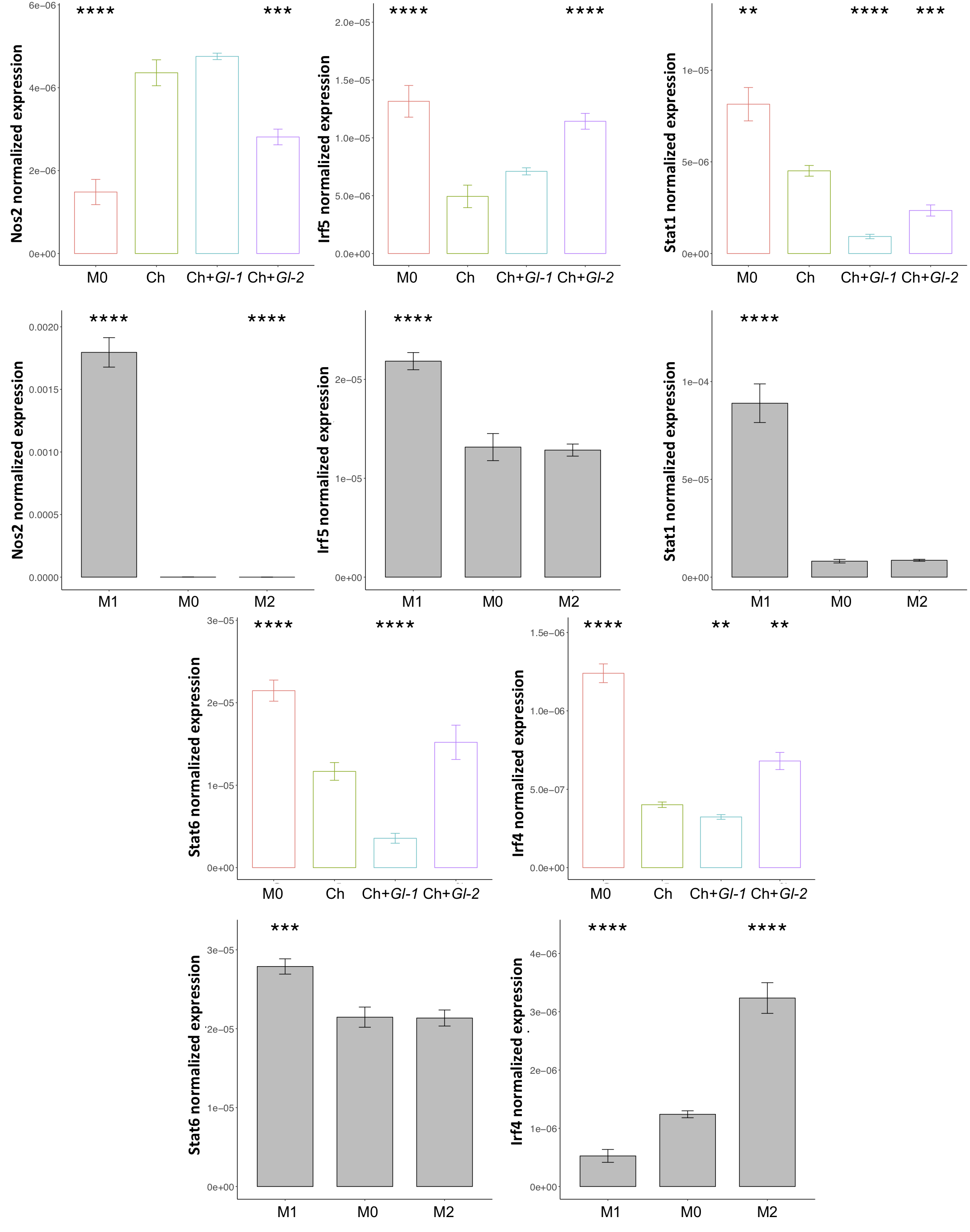

Supplement: Supplementary file 1 [file nutrients-13-00038-s001.zip › Supplmentary/SupFig5.jpg]

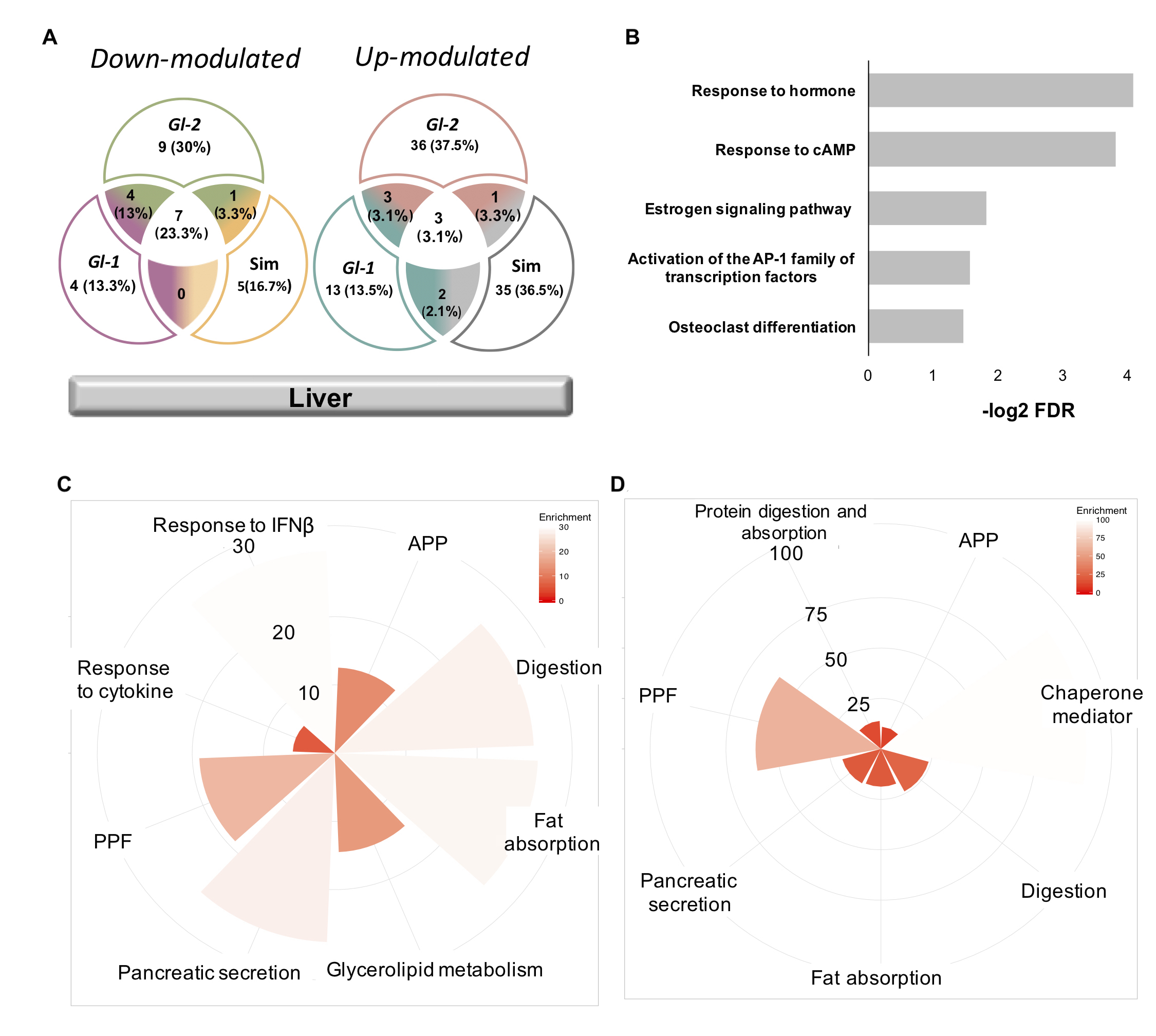

Supplement: Supplementary file 1 [file nutrients-13-00038-s001.zip › Supplmentary/SupFig4.jpg]

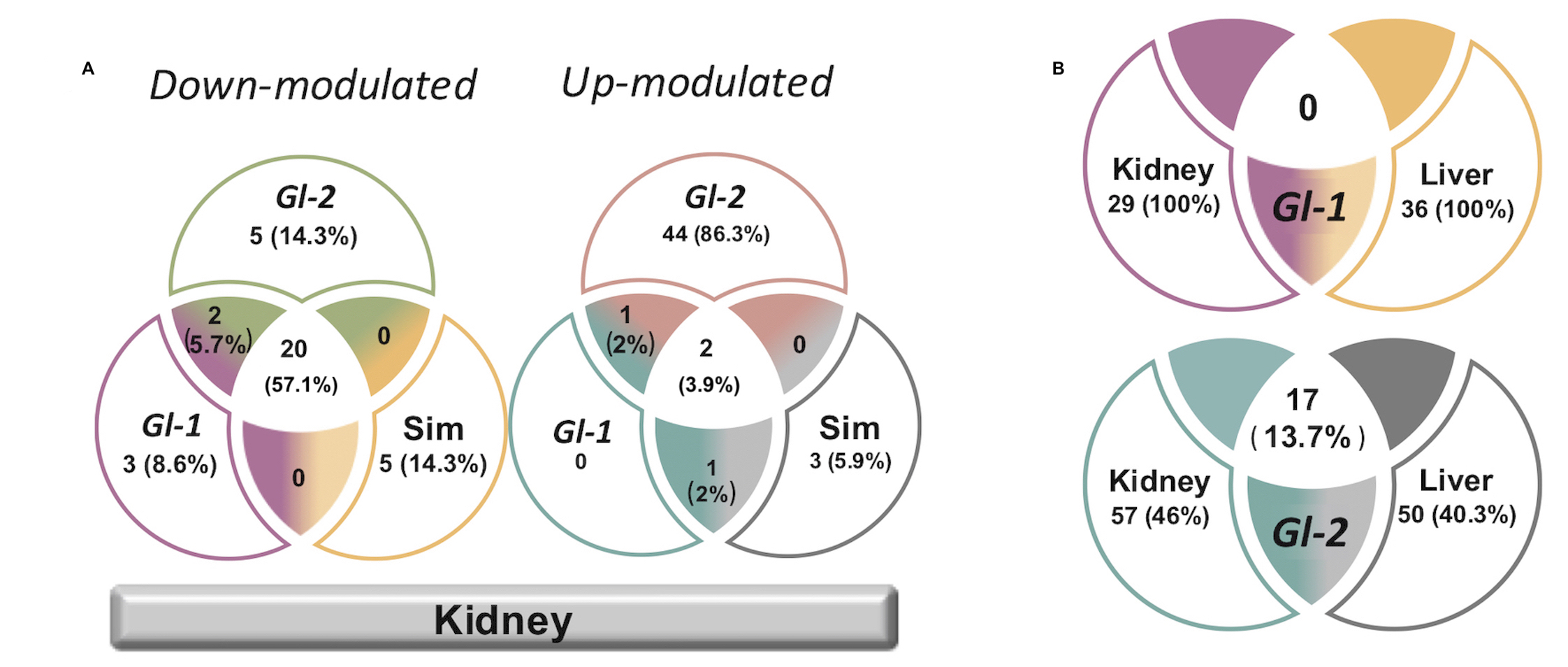

Supplement: Supplementary file 1 [file nutrients-13-00038-s001.zip › Supplmentary/SupFig6.jpg]

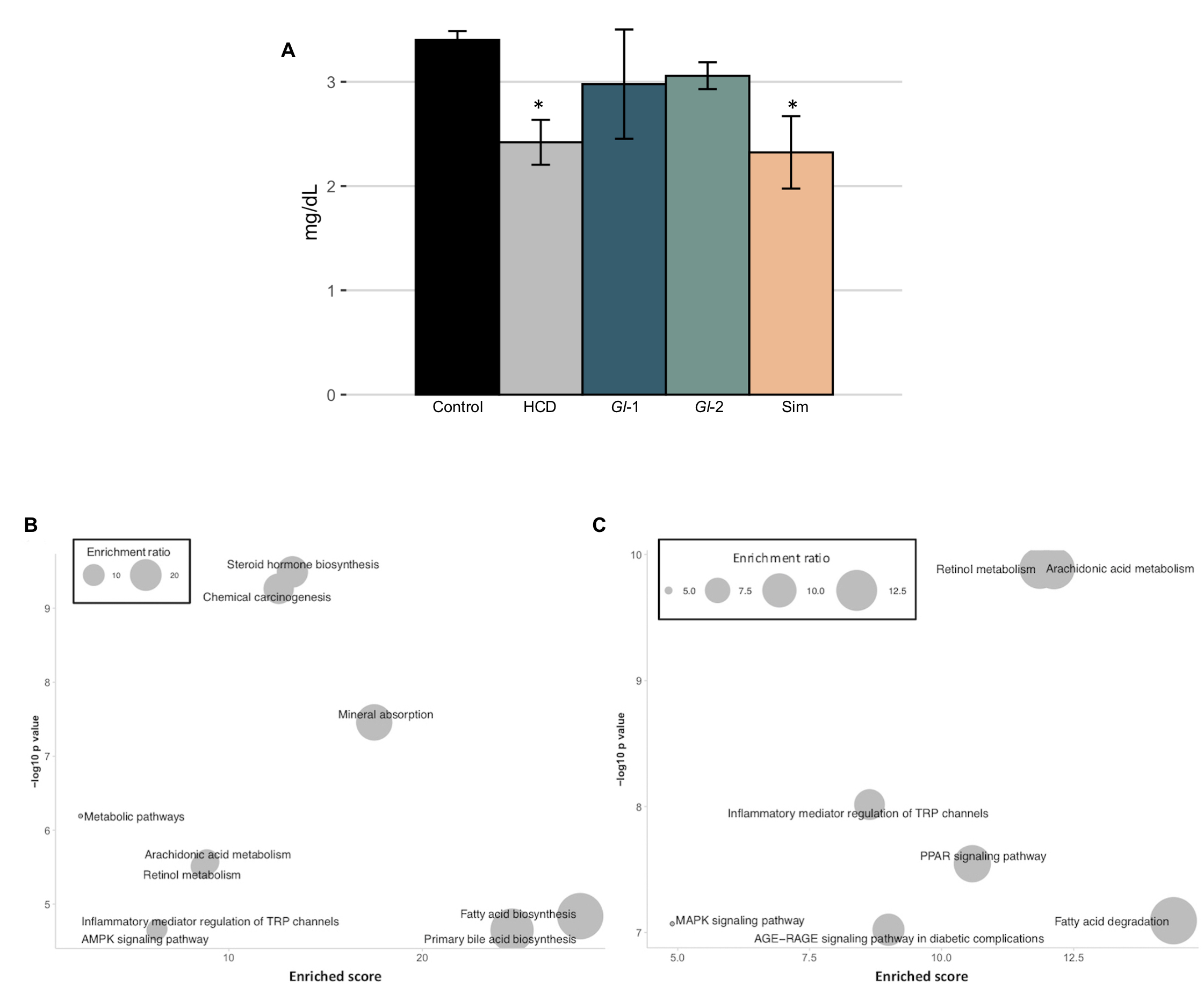

Supplement: Supplementary file 1 [file nutrients-13-00038-s001.zip › Supplmentary/SupFig7.jpg]

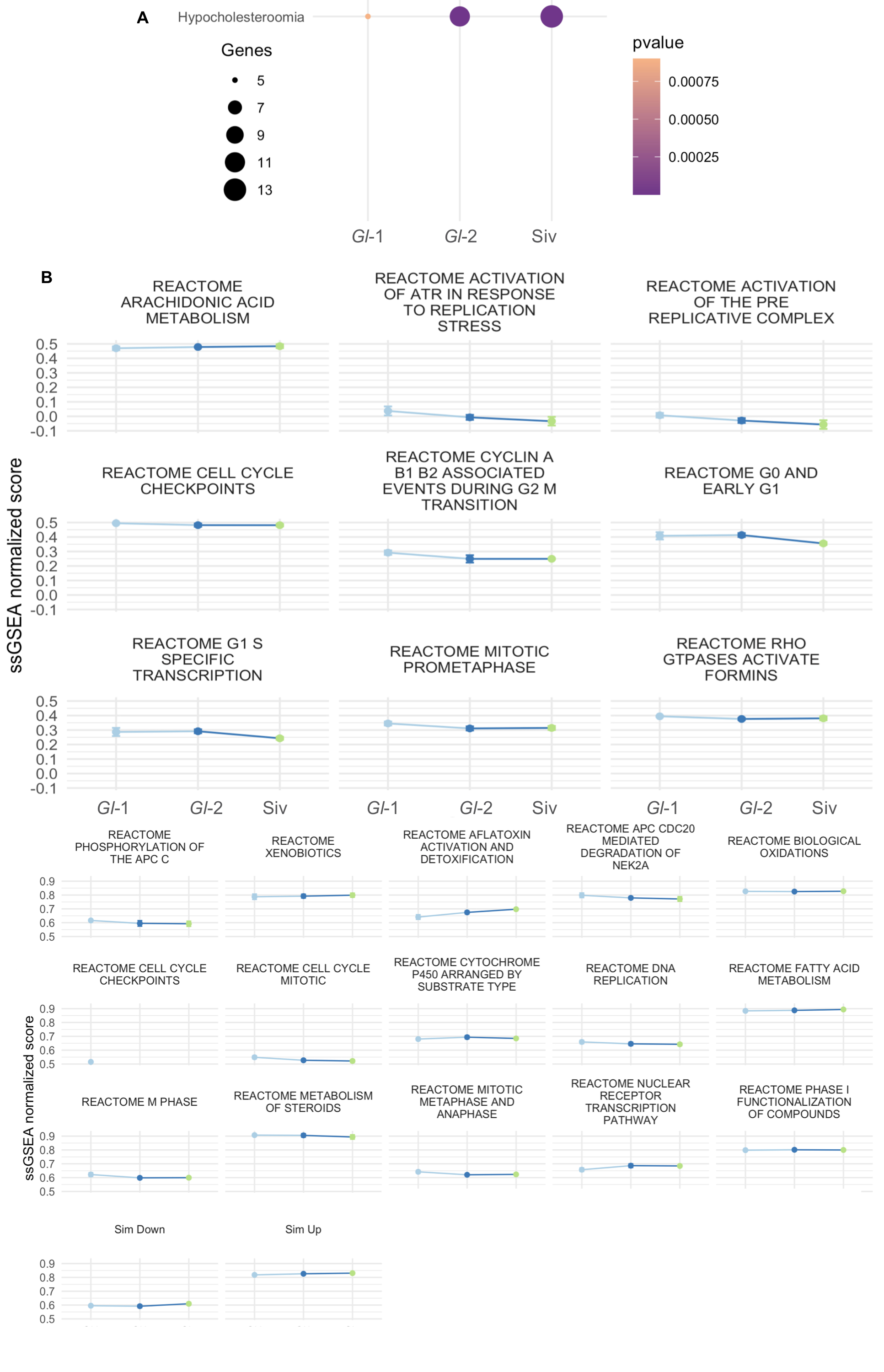

Supplement: Supplementary file 1 [file nutrients-13-00038-s001.zip › Supplmentary/SupFig3.jpg]

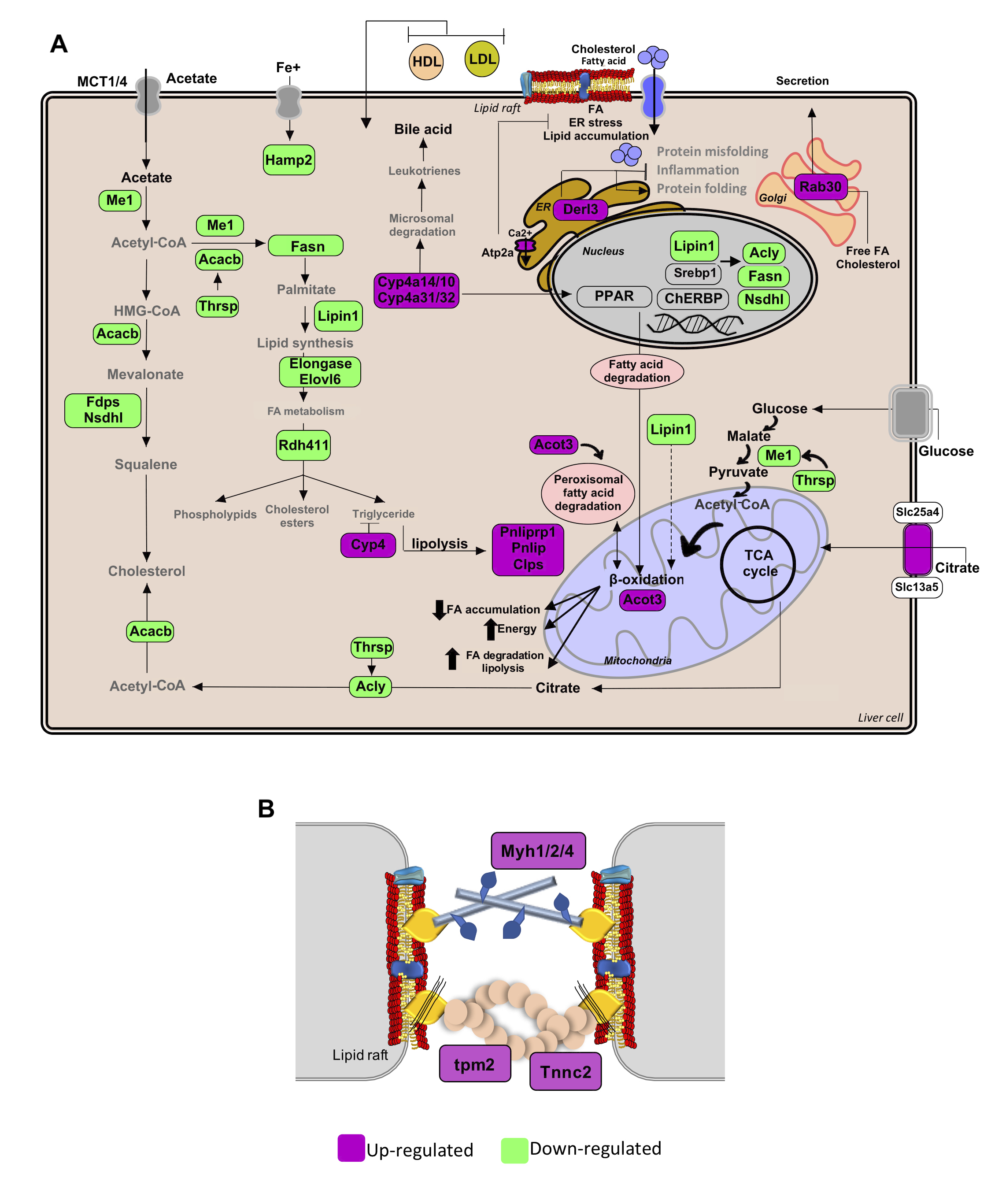

Supplement: Supplementary file 1 [file nutrients-13-00038-s001.zip › Supplmentary/SupFig2.jpg]

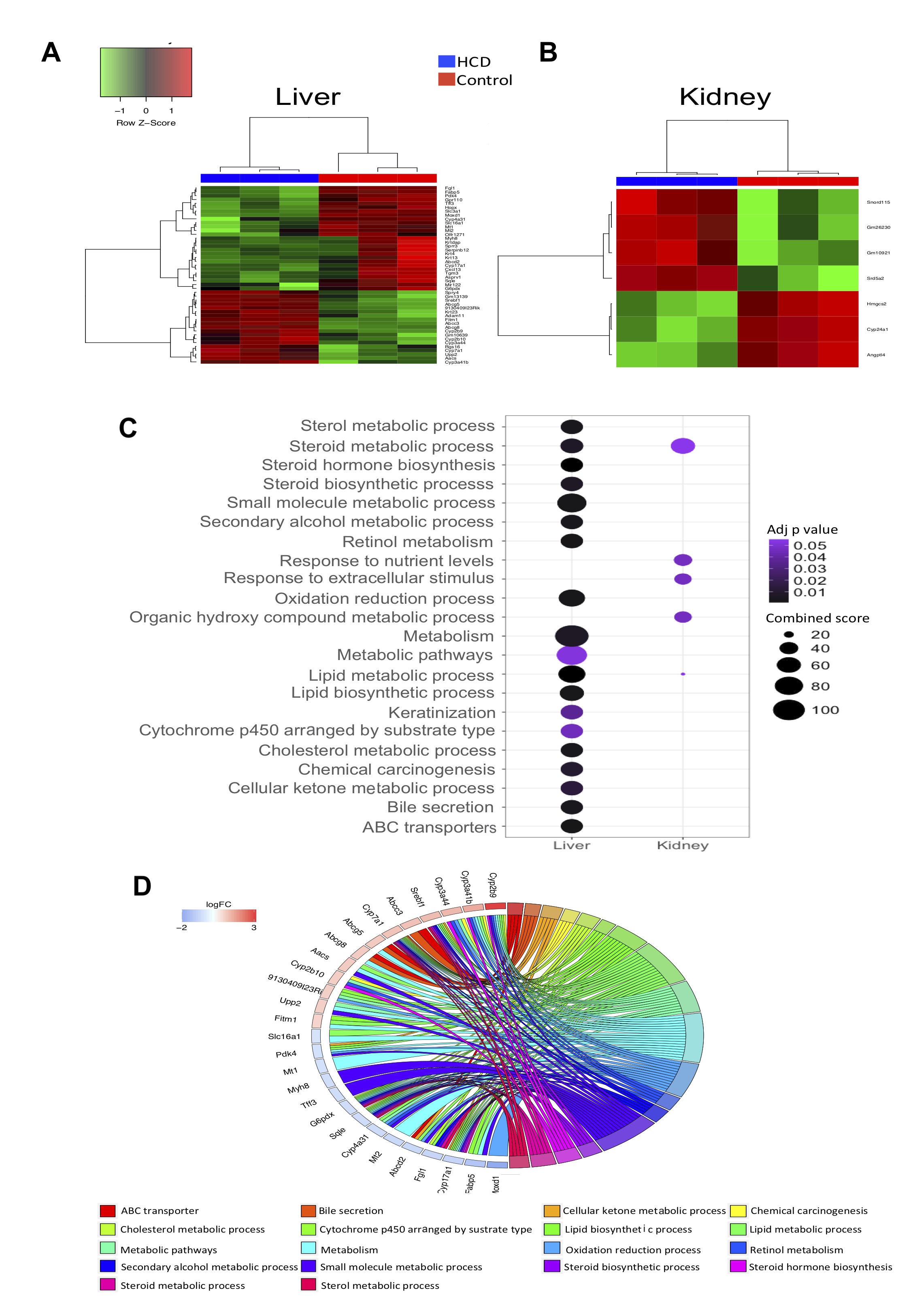

Supplement: Supplementary file 1 [file nutrients-13-00038-s001.zip › Supplmentary/SupFig1.jpg]
